# Statistical Considerations on the Use of RWD/RWE for Oncology Drug Approvals: Overview and Lessons Learned

**DOI:** 10.1007/s43441-023-00528-y

**Published:** 2023-05-13

**Authors:** Sunhee K. Ro, Weidong Zhang, Qi Jiang, Xiaoyun Nicole Li, Rong Liu, Chengxing Cindy Lu, Olga Marchenko, Linda Sun, Jing Zhao

**Affiliations:** 1 Sierra Oncology Inc: GlaxoSmithKline Inc, San Mateo, USA; 2 Sana Biotechnology Inc, Seattle, USA; 3 Seagen Inc, Bothell, USA; 4 BeiGene Ltd, Ridgefield Park, USA; 5 Bristol Myers Squibb Co., New York, USA; 6 AstraZeneca Plc, Cambridge, UK; 7 Bayer, Barmen, Germany; 8 Merck & Co. Inc, New York, USA

**Keywords:** Real world data, Real world evidence, Oncology drug approval, Clinical study design, Control arm augmentation

## Abstract

Despite increasing utilization of real-world data (RWD)/real-world evidence (RWE) in regulatory submissions, their application to oncology drug approvals has seen limited success. Real-world data is most commonly summarized as a benchmark control for a single arm study or used to augment the concurrent control in a randomized clinical trial (RCT). While there has been substantial research on usage of RWD/RWE, our goal is to provide a comprehensive overview of their use in oncology drug approval submissions to inform future RWD/RWE study design. We will review examples of applications and summarize the strengths and weaknesses of each example identified by regulatory agencies. A few noteworthy case studies will be reviewed in detail. Operational aspects of RWD/RWE study design/analysis will be also discussed.

## Introduction: Overview of Value of RWE in Oncology Drug Development

While RCTs are the established gold standard in clinical drug development, conducting large- scale RCTs is not always possible [1], especially in rare disease settings. RWE derived from RWD holds great potential to increase efficiency and improve clinical development of new treatments in such cases. Healthcare data collected in actual clinical practice can also be a valuable resource for understanding the effectiveness and safety of a treatment [2]. As such, the use of RWD/RWE to support regulatory approval has been gaining increased attention in recent years. It was acknowledged that confirmatory evidence for the effectiveness of a drug could include RWD [3]. However, their use has yet to be widely accepted by regulatory agencies for various reasons. To our best knowledge, successful examples of RWE being used in oncology regulatory submissions are still limited and RWE is mostly still used as supportive evidence aiming to support the direct comparison, to isolate the treatment effect, or to establish the natural history of disease. We didn’t find any pivotal study in oncology that successfully supported a marketing application to the U.S. Food and Drug Administration (FDA) or the European Medicines Agency (EMA) in which the primary analysis included a formal comparison using RWD [1] although there are a few such examples that led to FDA approval in non-oncology settings (e.g., Omegaven, [4]).

Understanding the key issues and concerns raised by regulatory agencies is an important first step to addressing these issues and eventually paving the way to the successful use of RWD/RWE for regulatory approvals. In this manuscript, we will provide a summary of the key issues and concerns raised by regulatory agencies regarding the use of RWE to support marketing applications in oncology (Sect. “Key Issues and Concerns with RWD/RWE Raised by Health Authorities in Terms of Regulatory Approval Consideration”). Examples with both successful and unsuccessful outcomes will be summarized in the same section. Definition of RWD, RWE and different types of RWD will be given below in this section. In Sect. “Role of RWD and RWE in RCTs”, we will focus on the roles of RWD and RWE in RCTs and present a promising case study of a recently designed randomized study with both concurrent and external control arms using RWD that received positive FDA feedback. A few examples for using RWD/RWE will be described in detail in Sect. “Case Studies” to provide a closer look at the issues from a case with unsuccessful outcome as well as introduce examples of good practices. Recommendations on the use of RWD to successfully generate evidence for the comparative effectiveness of treatment in oncology studies will be provided. The goal is to provide best practices for using RWD for comparative treatment effect estimates.

Throughout the manuscript, we will use the following definition of RWD and RWE based on the definitions in the FDA draft guidance [5] except that our definition of RWD also includes data from non-contemporaneous controls from other clinical studies.

- RWD is data relating to patient health status or the delivery of health care routinely collected from a variety of sources ***or clinical study data external to the study of the experimental arm.***
- RWE is the clinical evidence regarding the usage and potential benefits or risks of a medical product derived from the analysis of RWD.

Sources of RWD can be grouped into two broad categories ([2, 6])

(1) Category I: Research studies, including historical clinical study data and nonexperimental research data

Category I data are initially collected for clinical research. The strength of such data is that the primary research questions that lead to the data being collected are well-defined [7]. Ideally this leads to explicit inclusion/exclusion criteria defining the study population and explicit definitions of treatment variables, outcome variables, as well as any key confounders related to the outcomes. This knowledge can be used to determine if the data are suitable for an RWE study under consideration [2]. Actual examples of Category I RWD include the data from a systematic literature review of efficacy from the standard of care treatment for refractory diffuse large B-cell lymphoma (rDLBCL) which was used to compare the single arm result of polatuzumab vedotin-piiq submission to for FDA submission. Another example also related to rDLBCL is the SCHOLAR-1 study which is a pooled meta-analysis cohort of 2 clinical studies and 3 observational studies as external control for the review of Kymriah ([8], more details in Sect. “Case Studies”).

(2) Category II: “transactional RWD” from the operation of healthcare systems

In contrast to category I, the initial purpose of category II data collection is not for clinical research, but for clinical documentation or administrative purposes. Electronic health records or medical claims data belong to this category. Examples of Category II oncology RWD database are shown in Table 1 below.

**Table 1:** Oncology Database Examples of Category II RWD Sources

Source type	Database example
Electronic health records (EHR)	•Flatiron health analytic database •iKnowMed EHRs •Large academic medical centers, e.g., MD Anderson •EHR-derived de-identified Flatiron Health-Amgen OSCER database
Medical claims data	•IQVIA pharmacy and medical claim data
Patient registry	•European bone marrow transplant (EBMT) registry •The SEER research data (NCI)* •National cancer registration and analysis service (NCRAS) UK

*SEER-linkage (SEER-medicare and SEER-medicaid etc.) is hybrid type database which aims to link registry and medical claim databases

Electronic Health Records (EHR): are electronic versions of patients’ medical history, maintained by the provider over time and used by medical practices and facilities to care for patients. The strengths of EHR are that the data frame is broad, and it includes more diverse research questions to understand clinical management of diseases over time. Another strength of EHRs is that it may be connected with genomic database, e.g., Flatiron and Foundation Medicine’s clinic-genomic database, for the advancement of personalized cancer care. EHRs contain structured fields (such as demographics, medical history, diagnoses, and prescribed medicines) and unstructured fields (such as health provider notes including reasons for uptake and discontinuation of treatments). Although EHRs provide benefits by capturing clinical detail on patients over time, they are often restricted to care received from a specific provider or network of providers [9]. An example of EHR data that was used to support regulatory submission in oncology is iKnowMed EHRs, which were used as an external control to single arm data of Avelumab to support accelerated approval submission of Avelumab as treatment for merkel cell carcinoma to FDA. The FDA commented that the data were limited and subject to selection bias [10].

Medical Claims Data: are administrative claim databases that are used in the administration and billing of health care organizations, such as healthcare insurers. The strength of Medical Claims data is that the data are collected in a longitudinal nature, and often includes information on diagnoses, procedures, and medicines dispensed such that it may facilitate answering questions on disease prevalence, incidence, burden of illness, treatment pattern, or disease progression [2]. Because of the repurposed use of administrative claims data into clinical research setting as RWE, the completeness and relevance of claim data may be less clear and subsequent validation studies are likely to be required. Missing data and potential bias in data collection are the main drawbacks of using administrative data. The example of palbociclib US label expansion to include male patients with hormone receptor (HR)-positive, human epidermal growth-factor (HER) 2-negative advanced or metastatic breast cancer represents one of the first instances where medical claims data were utilized as RWD to support a regulatory decision in a rare oncology patient population. In this example, IQVIA pharmacy and medical claim data supported research on the duration of treatment for men with the above disease, while the Flatiron EHR supported real world response rate for men treated with Palbociclib [11].

Registry Records: Cancer registries collect data to (1) identify emerging cancer trends to understand contributing factors, (2) investigate health disparities in cancer incidence, prevalence, mortality, and survival, (3) understand patterns of care in the cancer patient population, and (4) study the impact of early detection and treatment advances on cancer incidence and outcomes(https://seer.cancer.gov/registries/cancer_registry/research_application.html). Registry data may share similar strengths and limitations as EHRs and medical claims data due to its administrative nature. Levenson et al. [2] pointed out that randomization can be performed within a disease or medical project or a health system which can provide a source of potential trial participants and associated clinical and health data [12].

It should be noted that there are additional challenges interpreting and handling data “missingness” for Category II RWD sources compared to data from clinical trials. Unlike clinical trials in which data is collected per the scope and schedule pre-specified in order to address particular clinical questions of interest, EHR data are usually collected when patients have medical encounters thus it may be more difficult to determine/interpret the amount, pattern and its impact on data analysis of missing data with EHR data [13].

## Key Issues and Concerns with RWD/RWE Raised by Health Authorities in Terms of Regulatory Approval Consideration

While there are various types of study designs utilizing RWD, such as randomized designs using both traditional clinical study elements and RWD, the most widely used designs utilizing RWD for regulatory submission are those that incorporate RWD as an external control. This section will focus on major issues and concerns raised by health authorities with the designs using this approach.

When it comes to the use of RWD as external control, it is critical to answer the following two questions:

1. What is the quality of the RWD and can the study used to generate the RWD provide adequate scientific evidence to answer regulatory questions of interest?
2. What are the benefits and limitations of using RWD given the study setting? Can such limitations be properly addressed and accounted for?

To address both questions, the FDA developed a framework in the 21st Century Cures Act [14] for evaluating the quality and relevance of RWD/RWE and summarized it as a three-part approach, aiming to incorporate RWE into regulatory decision making in evaluating individual supplemental applications. The EMA also published an operational, technical and methodological framework for regulatory use of RWE to address these questions and provided possible solutions to the issues identified [15]. Other agencies such as the Pharmaceuticals and Medical Devices Agency (PMDA) and Medicines and Healthcare Products Regulatory Agency (MHRA) also published documents ([16–18]) addressing similar points, stating that data reliability and the appropriateness of the methodology for analysis are the major discussion points in utilizing RWD and RWE for regulatory decision making.

The first question focuses on the reliability and relevance of the data to the question of interest, which can directly impact the feasibility of using RWD to establish evidence for comparative effectiveness. An answer to this first question should include whether the RWD includes necessary data on exposure, outcome, and covariates and also whether the collected data format is adequate. For example, EHR and medical claims may include major clinical events, but not all that is required. The structure of captured data in these data sources might be loose and inadequate for analysis. This issue is of tremendous importance and directly impacts the feasibility of using RWD to establish comparative effectiveness. As we can see in the examples below, many trials failed to provide adequate evidence due to missing covariates, different assessment methods for the primary endpoint, etc. In addition, the draft FDA RWE guidance [5] recommends that the appropriateness and limitations of the data source for the study question should be addressed in the protocol.

When answering the second question, keep in mind that using RWD and RWE is a sensible approach in some scenarios (as discussed in the previous section). For example, when a RCT is not possible to conduct, single arm studies are typically considered as an alternative. However, a single arm study alone might not be enough to establish the benefit of the experimental treatment and the use of data external to the single arm data can be considered to supplement it for estimating comparative effectiveness. Additionally, the use of RWE can also be appealing when the experimental treatment is intended to treat a rare and serious disease, for which (a) the disease course is well-documented, highly predictable, and can be objectively measured, (b) the study population and external controls are suitably comparable, and (c) the external treatment effect is large, self-evident, and closely associated temporally with the treatment intervention.

When a particular piece of RWE is based on other clinical study(s) to serve as external control for a single arm pivotal study, some critical regulatory concerns exist over the potential bias the RWE may incur, including confounding bias, selection bias, temporal bias, as well as unobserved bias. Such biases reduce one’s confidence that the observed differences in efficacy or safety outcomes can be attributed to the investigational intervention in the absence of randomization. Although the idea of accounting for the potential bias from the use of RWD as external control has been heavily researched [2], best practices for study design and data analysis strategies in this setting have yet to be defined and, therefore, challenges still exist.

Failure to adequately address these two questions above has led to many unsuccessful proposals to use RWE to support the marketing application. We identified several published studies in oncology where external control data were used to establish comparative effectiveness that were submitted to or evaluated by the FDA and the EMA for regulatory approval (Table 2). Comparisons using RWD (external control) play a supportive, rather than pivotal, role in most of the cases listed. Most of submitted evidence was deemed not adequate to establish comparative effectiveness with the most common reasons for inadequacy being: (1) uncollected confounding variables or too much missing data in the important confounding covariates; (2) small sample size of the matched set (all by propensity score matching); (3) different assessment method for primary endpoint between the study data and RWD; and, (4) primary endpoint appropriate for non-randomized comparison mostly limited to response rate rather than time to event endpoints, among others.

**Table 2:** Examples of Using External Control for Comparative Effectiveness with Regulatory Purpose in Oncology

Drug	Selinexor [21]	Tafasitamab [22, 23]	Blincyto[24]	Erdafatinib [25]	Pembro/Lenvatinib[26]	Various PD-L1/PD1 drug combinations [27]	Yescarta [31]	Kymriah [8, 15]
Disease	RRMM	DLBCL	PhALL	Unoperable/metastatic Urothelial Cancer with FGFR alterations	Endometrial carcinoma	Metastatic RCC	RR DLBCL	RR DLBCL
Objective	Comparing Selinexor monotherapy single arm study to RWD as external control	Comparing Tafa/Len combo single arm study to RWD Len monotherapy to isolate the added benefit of Tafa	Comparing single arm blincyto study to historical control to demonstrate no harm of Blincyto treatment	Comparing single arm Erdafatinib study to RWD PD-L1/PD-1 data	Comparing the signle arm Pembro/Lenvatinib combo study to the historical studies with Pembro mono or lenvatinib mono to isolate each drug’s effect	Comparing monotherapy efficacies of each combo drugs to sunitinib to isolate their contribution in the combo drug efficacy	providing confirmation of the pre-specified control response rate and context for interpreting the single arm study results	providing confirmation of the pre-specified control response rate and context for interpreting the single arm study results
Endpoint used in comparison (primary/other)	OS	ORR/CRR/PFS/OS	MRD negativity rate rate/RFS/OS	OS	ORR	ORR	ORR	ORR
Proposed as primary or supportive evidence	Supportive	Supportive	Supportive	Supportive	Supportive	Supportive	Supportive	Supportive
Proposed for initial or secondary indications	Initial	Initial	Revision of initial indication	Initial	Secondary***	Secondary***	Initial	Initial**
Major approach for adjusting bias due to lack of randomization	PS matching	PS matching		PS based weighting and stabilized IPTW. Delayed entry model	PS based comparison and sensitivity analysis across multiple unmeasured confounder values*	PS matching	Standardized method (by EMA), PS matching method (by FDA)	PS based MAIC
Major issue (identified by regulatory agencies)	Selection bias due to I/E discrepancies, immortal time bias due to the incompatible index definition, operational bias of not pre-specifying the analysis	Small sample size, missing data, discrepancies in endpoint evaluation		Missing data, small sample size	Not available	Not available	Not available	Modest improvement (of ORR) over most relevant historical control study
Factors mitigating issues/strengthening RWE					Magnitude of improvement in combination over monotherapy controls, consistency in efficacy between overall and matched subset	Consistency efficacy between overall and matched subset of the combination study, OS benefit of the combination over control (sunitinib) in the primary RCTs, strong biological rationale	Consistent efficacy across different subgroups of the single arm study, magnitude of improvement in the single arm study over historical controls	Magnitude of improvement in Duration of response over historical controls
Outcome by regulatory agencies	Not able to demonstrate evidence for comparative efficacy	Not able to demonstrate evidence for comparative efficacy			Demonstrated evidence for added benefit of each drug in combination therapy	Demonstrated evidence for added benefit of each drug in combination therapy	Demonstrated evidence for the benefit of the single arm study result to historical control	Demonstrated evidence for the benefit of the single arm study result to historical control

*RR* Relapsed Refractory, *MM* Multiple Myeloma, *DLBCL* Diffuse Large B-cell Lymphoma, *PhALL* Philadelphia chromosome-positive acute lymphoblastic leukemia, *FGFR* fibroblast growth-factor receptors, *RCC* Renal Cell Carcinoma, *PD-L1* Programmed death-ligand 1, *PD-1* Programmed cell death protein 1, *IPTW* Inverse probability of treatment weighting, *PS* Propensity Score, *MAIC* Matching adjusted indirect comparison, *ORR* Overall response rate, *CRR* Complete response rate, *PFS* Progression free survival, *OS* Overall survival, *MRD* Minimal residual disease, *RFS* Relapse free survival, *I/E* Inclusion/exclusion, *Tafa* Tafasitamab, *Len* Lenalidomide

*Analysis done by FDA [26], **In adult population, ***After monotherapy indications

However, there were a few successful examples which led regulatory agencies to acknowledge the benefit of using an external control, although the RWD is still supportive. In the FDA’s own analysis, cross-trial comparison between the single arm Pembro/Lenvatinib and each drug’s monotherapy studies in endometrial carcinoma helped to isolate each individual drug’s contribution to the combination therapy’s efficacy. Various anti Programmed cell death protein 1 (PD-1)/ Programmed death-ligand 1 (PD-L)1 drug combination therapy arms from the RCTs against Sunitinib were compared against each component’s monotherapy studies via cross-trial comparisons and resulted in a successful outcome [19]. Nevertheless, it should be noted that the OS advantage observed in the combination therapy vs the Sunitinib monotherapy RCT contributed to the overall conclusion that each component has an added benefit to the combination, in addition to the RWE used. Other examples of RWD being used successfully are found in the approvals of Yescarta and Kymriah. The primary study for their approval as the treatment of relapsed or refractory diffuse large B-cell lymphoma (RR DLBCL) after two or more lines of systemic therapy is a single arm study (ZUMA-1) which compared the treatment response rate with a pre-specified response rate based on literature. In order to provide context to this comparison, a patient-level pooled analysis of a population chosen to match the ZUMA-1 population was conducted ([8, 20]). The details of these examples are described in Sect. “Case Studies”.

From the examples of successful cases, one can observe that the magnitude of improvement in the treatment arm over the RWD (external control) is crucial to strengthening the RWE from the cross-trial comparison, and in making its value more likely to be acknowledged by regulatory agencies. Consistency across different subgroups and/or between the overall population and the matched subset of the experimental arm is also shown to strengthen the RWE.

A pre-specified Statistical Analysis Plan (SAP) using RWD is critical. The SAP should include statistical methods to account for various types of potential bias. The suitability of those methods should be justified. In addition, the SAP should include a formal assessment of the similarity of patient populations between arms using pre-specified balance criteria, both before and after any statistical adjustment procedures are applied and prior to the analysis of outcome data. Comprehensive sensitivity analyses should be conducted especially when the statistical adjustment procedures involve unverifiable assumptions. It is prudent to have a firewall between statisticians working on population matching and those working on treatment effect estimation. For all studies using EHRs or medical claims data that will be submitted to the FDA to support a regulatory decision, protocols and statistical analysis plans should be submitted before conducting the study [4].

## Role of RWD and RWE in RCTs

Even when a RCT is feasible and conducted, there are still situations where RWD can add value. As stated, single arm studies that use an external or historical control are associated with a higher risk of bias and are difficult to assess. In comparison, in RCTs where analysis is conducted with a combination of concurrent and external controls, the use of RWE provides an opportunity to increase the study’s power, reduce variability, and control the risk since the fallback plan could always be only using the randomized control if randomized control and external control differ. Pocock [28] summarized that in order for historical controls to be acceptable to be used as part of a randomized trial, the following conditions are required for the data: 1) the patients in the data must have the same treatment as the randomized controls; 2) the data must have had same patient eligibility criteria with comparable distribution of important baseline characteristics; 3) the methods of treatment evaluation must be the same; 4) it must have been performed in the same organization as the RCT with largely the same clinical investigators; 5) there must be no other indications leading one to expect differing results between the randomized and historical controls. It will be difficult for RWD from other data source to meet these requirements. Historical control data from previous clinical trials will have higher chances of meeting these criteria and provide higher quality of external control cases ([29, 30]). Important applications of RWD in RCTs include augmenting control arm in a RCT to expedite drug development which is especially meaningful for diseases with high unmet medical needs such as recurrent Glioblastoma (GB) (NCT 01582269, [32]). An external control can also be used in a RCT to increase power for a long-term treatment effect where the existing RCT can only be fully powered for a relatively short-term endpoint, and not for the long-term clinical endpoint. One recent example for this is the complex innovative design (CID) by Roche in DLBCL, with PFS as the registrational endpoint but reasonably powered OS analysis is still required by regulatory agency which will prolong the study due to increased sample size/longer follow-up. In this case, using reliable external data is preferred over enrolling additional patients with extended follow-up in existing RCTs [33]. Utility of external study for the DLBCL case will be elaborated later in Sect. “Case Studies”.

Burger et al. [34] and Ghadessi et al. [35] provided detailed summaries and recommendations on the use of external controls. When introducing external controls, bias could be introduced. It is crucial for the sponsor to take external control into careful consideration with regards to the time trend, population, change of standard of care, logistics, and other risk factors to have a clear understanding of the disease, detailed characteristics of the affected population, precise definition of the endpoint and clear definition of the diagnosis in terms of what and how it has to be measured to minimize the potential bias when using RWD in RCTs.

Methodology for borrowing RWD to identify control arm treatment effect is another important aspect of RWD application in RCT trials and we have seen rapid development in this area in recent years ([36–38]). There are two types of borrowing, static borrowing and dynamic borrowing. Bayesian methods on data borrowing as well as two-stage methods with both propensity score adjustment and Bayesian borrowing have been proposed [36]. Dynamic borrowing is usually preferred as it is difficult to predict how similar or different the external data would be to the clinical trial of interest. However, it is challenging to determine the choice of prior weight for the informative prior when using Bayesian dynamic borrowing. Sensitivity analyses or tipping point analyses should be conducted using a range of priors to evaluate the robustness of the results [37].

## Case Studies

Despite the concerns and challenges mentioned earlier, there is great potential for using RWD and RWE as primary or strong supportive evidence for regulatory approval in pivotal trials. In this section, we present details of a few case studies involving RWD and RWE which received favorable outcomes from regulatory agencies either in the design of the study or evidence it generated. The goal is to have a closer look at the issues and strengths of each example. In the first two case studies, subjects from ongoing external trials or historical trials were identified as external comparator arms for the treatment arm of interest, whereas in the third example external studies were used to develop benchmark comparators for single arm pivotal trials.

### Case study #1: External Control in a Phase 3 Study in 1L DLBCL

DLBCL is the most common non-Hodgkin’s lymphoma (NHL). The standard of care in first line DLBCL is R − CHOP immunochemotherapy. However, approximately 30–50% of DLBCL patients are not cured by this first line treatment [39]. To meet this unmet medical need, Roche proposed a randomized, open-label, multicenter trial in patients with first line DLBCL randomized 2:1 to treatment vs control with PFS as the primary and OS as the key secondary endpoint. In addition to this concurrent control arm randomized within this study, approximately 100 patients with control arm treatment from another ongoing study were also added as “external” control arm. The patients in the external control arm were selected using the propensity score matching method after applying similar inclusion/exclusion criteria of the two trials. For the planned OS analysis, a Bayesian commensurate prior with a Weibull model [38] was proposed to dynamically borrow information from the external control arm. Using the data from the external control arm has the clear advantage of a reduced number of patients required in the concurrent control arm. This design was reviewed by the FDA under the Complex innovative trial designs (CID) program due to its novelty and the complexity of the information borrowing from an external control and the use of a Bayesian parametric model for the secondary endpoint. As mentioned in the previous sections, the FDA focuses on certain considerations while reviewing the proposed design, such as (1) the comparability of the external trial and the trial of interest; (2) whether the design is robust to various model assumptions; (3) whether the proposed matching and borrowing methods are appropriate and interpretable; and (4) whether the type I error is well controlled considering various plausible deviations from the model assumptions. To address these considerations, thorough simulations were conducted to evaluate the operating characteristics (OC) of the proposed model that include (a) power and Type I error under differences in OS curve behavior between the trial control arm and external control arm and (b) the OC and performance of the model under deviations from the various model assumptions in terms of similarity in patient populations, survival time distribution, unmeasured confounding, and the linear form of the propensity score model etc. To increase the similarity of patient populations, the Sponsor plans to prioritize enrolling patients in the same sites for both the randomized arms and external control arm when possible. [33]. The inclusion of the external control data as part of the RCT design under CID program has highlighted potential benefit to increase the efficiency of trial design, thus has accelerated drug development process.

### Case study #2: Comparing Results Across Trials in Combination Drug Development

Combination drug regimens have been of great interest to drug developers who aim to identify their improved effect compared to monotherapy. For the regulatory approval of combination regimens, it is important to demonstrate the contribution of each monotherapy to clinical benefits and safety. Factorial designs are effective and unbiased in demonstrating the effect of combination therapy, though, factorial designs require large numbers of patients to be assigned in the potentially less efficacious monotherapy arms, and thus may be less preferred, and may take longer time to enroll patients. An alternative approach is to combine trials that have been conducted separately, e.g., trials of combination therapy or trials with single-agent therapy, and compare data collected across trials. Cross-trial comparisons are challenging due to discrepancies between trials such as patient population, clinical practices, and schedule of data collection etc. However, when properly planned, cross-trial comparison can be used to efficiently demonstrate combination drug effect, and lead to accelerated development and approval of combination regimens.

A few recent FDA approvals have demonstrated the value of the cross-trial comparison approach for combination drug development, as shown in two examples in Table 2. Here we would like to focus on the example of three combination regimens with anti-PD1 inhibitors in previously untreated, locally advanced or RCC approved by FDA [19]. The three combinations were nivolumab in combination with ipilimumab, pembrolizumab in combination with axitinib, and avelumab in combination with axitinib. As summarized in Table 3, combination therapies were evaluated in the main studies, while the monotherapies were evaluated in the external studies conducted at different times. In these cases, each drug in the combination regimen was previously approved for oncology indications. For each case, results of ORR, PFS and OS from combination therapies were compared with results from external monotherapy studies. In each case, significant improvement of ORR was observed in the combination study as compared to each of the monotherapy studies except for CheckMate 214, in which the improvement was not statistically significant. In addition, statistically significant improvement of OS, PFS, or both have been observed, which provided strong evidence to support regulatory approvals.

**Table 3:** Examples of Using External Monotherapy Studies to Provide Evidence for Approval of Combination Therapies

Pivotal combination study	External monotherapy study
CheckMate 214 (Nivolumab + Ipilimumab in previously treated advanced RCC) [41]	Study CA209009 (nivolumab monotherapy in the first line metastatic RCC) [40]
	CheckMate 025 (nivolumab monotherapy in second line advanced RCC trial) [42]
	MDX010-11 (ipilimumab monotherapy for patients in the first line metastatic RCC (NCT00057889)
KEYNOTE-426 (Pembrolizumab + Axitinib in treatment-naïve advanced RCC) [43]	KEYNOTE-427(Pembrolizumab Monotherapy in first line locally advanced/metastatic RCC) [44]
	Axitinib (AG-013736) For the Treatment of Metastatic Renal Cell Cancer [45]
JAVELIN Renal 101 (Avelumab + Axitinib in treatment-naïve advanced RCC) [46]	Phase 1b JAVELIN (avelumab monotherapy first line treatment of patients with advanced RCC) [27]
	Axitinib (AG-013736) For the Treatment of Metastatic Renal Cell Cancer [45]

Although using external clinical data as a cross-trial comparison may introduce heterogeneity and uncertainty in general,, the improvement of multiple endpoints including ORR, PFS and OS, and strong biological rationale in these cases have provided robust evidence for the approval.

### Case study #3: Developing Benchmarks using patIent-level Analysis of Outcomes of Refractory DLBCL from 2 Large Randomized Trials and 2 Academic Databases

In 2017, the first Chimeric antigen receptor T cell (CAR-T) therapy, Kymriah, was approved by the FDA and subsequently the EMA for the treatment of certain pediatric and young adult patients with advanced leukemia. Since then, a few other cell and gene therapy (CGT) drugs including Kymriah, Yescarta and Breyanzi have been approved by FDA and EMA for the treatment of RR DLBCL. One of the challenges of demonstrating efficacy for those approvals was to establish appropriate controls since most of the approvals were based on single arm studies to accelerate the approval process due to the extreme unmet medical needs. As part of the submission package to the EMA, the sponsor included the pivotal phase 2 single arm Kymriah study (Juliet) and Yescarta study (Zuma-1) treating RR DLBCL, and patients from the historical database, SCHOLAR-1, to derive the benchmark efficacy estimates. SCHOLAR-1 was an international, multicohort retrospective research study evaluating clinical benefits in patients with refractory non-Hodgkin lymphomas (NHL), including DLBCL. A total of 636 patients from SCHOLAR-1 comprising the observational follow-up of two phase 3 clinical trials and two observational cohorts were extracted based on refractory criteria. A form of propensity score weighting as matching adjusted indirect comparison method was used to further adjust for cross-study differences in patient characteristics after ensuring the same inclusion/exclusion criteria were applied for both Juliet and SCHOLAR-1 in the Kymriah submission. ORR in the SCHOLAR-1 study was 26%. Both Juliet and Zuma-1 studies used an ORR ≤ 20% as their null hypothesis in their protocols. The observed ORR was 50% (95% CI:38%, 62%) and 72% (95% CI: 62%, 81%) from the Juliet [47] and Zuma-1 [48] studies, respectively. Both studies demonstrated improved efficacy in ORR when compared to the null hypothesis and the benchmark ORR derived from SCHOLAR-1 [8]. In addition to SCHOLAR-1, two other external datasets (the pooled CORAL extension study and PIX301) were included in the Kymriah submission to contextualize the results from the single arm Juliet trial. The unadjusted ORR from PIX301 was 30% and 40.3% from CORAL extension studies. Although the EMA commented in their assessment report that the CORAL study should be considered as the most relevant historical database for indirect comparison to the Juliet trial and that the efficacy was modest based on that benchmark efficacy, they considered the duration of response in complete responders to be substantial and clinically relevant in the patient population.

### Case study #4: Unsuccessful Use of RWD in Demonstrating Clinical Benefits of Exportin 1(XPO1) Inhibitor from a Single Arm Study

STORM was a multicenter, open-label, single arm registrational trial evaluating Selinexor, a XOI1 inhibitor, in combination with dexamethasone in patients with RRMM [21]. The ORR was 25.4% and the median DOR was 4.4 months. The Applicant also submitted an analysis of RWD, Study KS-50039 from the Flatiron Health Analytic Database (FHAD) in support of the NDA. However, the FDA concluded that 1) the analyses were not pre-specified and had methodological issues 2) patients between two studies were not comparable 3) direct comparison between two studies provided biased estimate of treatment effect in overall survival. Thus, the FDA concluded that the evidence generated from the RWD was insufficient to provide supportive evidence for the NDA submission. This example again highlights the importance of pre-specification of analysis, patient comparability and robustness of statistical methods in use of RWD as discussed from the previous sections.

## Discussion/Conclusion

There has been increasing utilization of RWE generated from RWD in regulatory submissions for oncology drug approvals, but successful cases are still limited. The unique concerns of RWE include the following: relevance of data to answer the questions of interest in terms of patient population, sample size of matched dataset, quality and completeness of RWD, unmeasured confounding and data missingness, and inconsistencies of endpoint evaluation methods which make the evidence generated from RWD more susceptible to bias. In most cases where RWE is used for regulatory submissions in oncology, RWE is generated by comparing the efficacy of an experimental treatment from an uncontrolled (usually single arm) study to that of a control treatment (often monotherapy components of an experimental combination treatment) from other studies, after dataset matching to adjust for confounding due to the lack of randomization.

In this paper, we gathered and reviewed recent cases of RWE being submitted for regulatory approval of oncology therapies in order to better understand major issues and strengths in their usage as identified by regulatory agencies. We observed that major issues identified in unsuccessful submissions included small sample size of matched datasets, large amount of missing data, and the magnitude of treatment effect not being sufficiently large enough to account for the added uncertainty of the comparative treatment effect estimated from RWE instead of a RCT. On the other hand, consistent efficacy between overall data and matched subset and/or across key prognostic subgroups and a large magnitude of treatment effect from RWE and strong biological rationale for the benefit of the experimental treatment over control strengthened the RWE, which resulted in successful outcomes in a few cases.

The usage of RWD to augment existing control arms in RCTs was reviewed separately (Sect. “Role of RWD and RWE in RCTs”) due to the method’s advantage of controlling the risk in the case that the RWD alone is very different from the population in a RCT. For this reason, control arm augmentation using RWD has been gaining substantial interest recently. We described several statistical methods of “borrowing” control arm effect from RWD to augment the control arm of existing study in this paper and described in detail a successful example of this design being accepted by regulatory in Sect. “Case Studies” (Case 1).

Operational aspects of RWD studies are also of great importance. The SAP should be developed prior to analyzing the endpoints. Key concerns of potential bias need to be addressed in the SAP and sensitivity analyses need to be considered alongside the sources of the RWD. A firewall between statisticians working on population matching (e.g., building propensity score model from data) and those working on treatment effect estimation is recommended. Careful thinking and planning from the very beginning of the study design is more needed in the design of a RWD study.

Future effort to generate RWE would benefit from more thoughtful thinking regarding challenges and potential resolutions, knowledge and real examples sharing, and more advanced methodological advancement in gaining broader applications in regulatory approvals.

## References

[1] Mishra-Kalyani PS. Statistical Considerations for External Controls in Pediatric Trials. Presentation at Meeting of Pediatric Oncology Subcommittee of Oncology Drug Advisory Committee. 2021.

[2] Levenson M, Weili H, Chen J, Fang Y, Faries D, Goldstein BA, Ho M, Lee K, Mishra-Kalyani P, Rockhold F, Wang H, Zink RC (2021). Biostatistical Considerations When Using RWD and RWE in Clinical Studies for Regulatory Purposes: A Landscape Assessment. Statistics in Biopharmaceutical Research.

[3] U.S. Food and Drug Administration. Demonstrating Substantial Evidence of Effectiveness for Human Drug and Biological Products. 2019.

[4] U.S. Food and Drug Administration. Omegaven Prescribing Information. 2018.

[5] U.S. Food and Drug Administration. Real-World Data: Assessing Electronic Health Records and Medical Claims Data to Support Regulatory Decision-Making for Drug and Biological Products. 2021. 10.1002/pds.5444 PMC9320939 35471704

[6] Franklin JM, Schneeweiss S (2017). When and how can real world data analyses substitute for randomized controlled trials?. Clin Pharmacol Ther.

[7] Haynes RB (2006). Forming Research Questions. J Clin Epidemiol.

[8] European Medicines Agency. Assessment report Kymriah.2018.

[9] Lin KJ, Schneeweiss S (2016). Considerations for the Analysis of Longitudinal Electronic Health Records Linked to Claims Data to Study the Effectiveness and Safety of Drugs. Clin Pharmacol Ther.

[10] Arondekar B, Duh MS, Bhak RH, DerSarkissian M, Huynh L, Wang K, Wojciehowski J, Wu M, Wornson B, Niyazov A, Demetri GD (2022). Real-world evidence in support of oncology product registration: a systematic review of new drug application and biologics license application approvals from 2015–2020. Clin Cancer Res.

[11] Kraus AL, Yu-Kite M, Mardekian J, Cotter MJ, Kim S, Decembrino J, Snow T, Carson KR, Motyl Rockland J, Gossai A, Wilner K (2022). Real-world data of palbociclib in combination with endocrine therapy for the treatment of metastatic breast cancer in men. Clin Pharmacol Ther.

[12] Fröbert O, Lagerqvist B, OlivecronaGK OE, Gudnason T, Maeng M, Aasa M, Angerås O, Calais F, Danielewicz M (2013). Thrombus aspiration during ST-segment elevation myocardial infarction. New Engl J Med.

[13] Goldstein BA, Phelan M, Pagidipati NJ, Peskoe SB (2019). How and when informative visit processes can bias inference when using electronic health records data for clinical research. J Am Med Inform Assoc.

[14] U.S. Food and Drug Administration. Framework for FDA’s Real-World Evidence Program. 2018.

[15] Cave A, Kurz X, Arlett P (2019). Real-world data for regulatory decision making: challenges and possible solutions for Europe. Clin Pharmacol Ther.

[16] Nishioka K, Makimura T, Ishiguro A, Nonaka T, Yamaguchi M, Uyama Y (2022). Evolving acceptance and use of RWE for regulatory decision making on the benefit/risk assessment of a drug in Japan. Clin Pharmacol Ther.

[17] MHRA guidance on the use of real-world data in clinical studies to support regulatory decisions. 2021. https://www.gov.uk/government/publications/mhra-guidance-on-the-use-of-real-world-data-in-clinical-studies-to-support-regulatory-decisions/mhra-guidance-on-the-use-of-real-world-data-in-clinical-studies-to-support-regulatory-decisions

[18] MHRA guidance on randomized controlled trials using real-world data to support regulatory decisions. 2021. https://www.gov.uk/government/publications/mhra-guidance-on-the-use-of-real-world-data-in-clinical-studies-to-support-regulatory-decisions/mhra-guideline-on-randomised-controlled-trials-using-real-world-data-to-support-regulatory-decisions

[19] Brewer JR, Chang E, Agrawal S, Singh H, Suzman DL, Xu J, Weinstock C, Fernandes LL, Cheng J, Zhang L, Xie D, Goldnerg KB, Bloomquist EW, Tang S, Sridhara R, Theoret MR, Pazdur R, Inrahim A, Beaver JA (2020). Regulatory consideration for contribution of effect of drugs used in combination regimens: renal cancer case studies. Clin Cancer Res.

[20] Neelapu SS, Locke FL, Bartlett NL, Lekakis LJ, Miklos DB, Jacobson CA, Braunschweig I, Oluwole OO, Siddiqi T, Lin Y, Timmerman JM, Stiff PJ (2017). Axicabtagene ciloleucel CAR T-cell therapy in refractory large B-cell lymphoma. N Engl J Med.

[21] U.S. Food and Drug Administration. 2019 Briefing Document for Oncology Drug Advisory Committee Meeting Selinexor NDA 212306

[22] U.S. Food and Drug Administration. 2020 Multi-discipline review and evaluation report Monjuvi (Tafasitamab-cxix) BLA 761173

[23] Zinzani PL, Rodgers T, Marino D, Frezzato M, Barbui AM, Castellino C, Meli E, Fowler NH, Salles G, Feinberg B, Kurukulasuriya NC, Tillmanns S, Parche S, Dey D, Fingerle-Rowson G, Ambarkane S, Winderlich M, Nowakowski GS (2021). RE-MIND: comparing Tafasitamab+Lenalidomide (L-MIND) with a real-world lenalidomide monotherapy cohort in relapsed or refractory diffuse large B-Cell lymphoma. Cancer Clinical Research.

[24] U.S. Food and Drug Administration. 2016 Statistical Review and evaluation report Blincyto BLA 125557

[25] U.S. Food and Drug Administration. 2019 Multi-discipline review and evaluation report, Erdafitinib NDA 212018

[26] Arora S, Balasubramaniam S, Zhang W, Zhang L, Sridhara R, Spillman D, Mathai JP, Scott B, Golding SJ, Coory M, Pazdur R, Beaver JA (2020). FDA approval summary: Pembrolizumab plus Lenvatinib for Endometrial Carcinoma, a Collaborative International Review under Project Orbis. Clin Cancer Res.

[27] Vaishampayan U, Schöffski P, Ravaud A, Borel C, Peguero J, Chaves J, Morris JC, Kotecki N, Smakal M, Zhou D, Guenther S, Bajars M, Gulley JL (2019). Avelumab monotherapy as first-line or second-line treatment in patients with metastatic renal cell carcinoma: phase Ib results from the JAVELIN Solid Tumor trial. J Immunother Cancer.

[28] Pocock SJ (1976). The combination of randomized and historical controls in clinical trials. J Chronic Dis.

[29] Viele K, Berry S, Neuenschwander BN, Amzal B, Chen F, Enas N, Hobbs B, Ibrahim JG, Kinnersley N, Lindborg S, Micallef S, Roychoundhury S, Thompson L (2014). Use of historical control data for assessing treatment effects in clinical trials. Pharm Stat.

[30] Ho M, Van der Laan M, Lee H, Chen J, Lee K, Fang Y, He W, Irony T, Jiang Q, Lin X, Meng Z, Mishra-Kalyani P, Rockhold F, Song Y, Wang H, White R (2021). The current landscape in biostatistics of real-world data and evidence: causal inference frameworks for study design and analysis. Stat Biopharm Res.

[31] European Medicines Agency. Assessment report Yescarta.2018.

[32] Smith CL, Thomas Z, Enas N, Thorn K, Lahn M, Benhadji K, Cleverly A (2020). Leveraging historical data into oncology development programs: Two case studies of phase 2 Bayesian augmented control trial designs. Pharm Stat.

[33] U.S. Food and Drug Administration. DLBCL Case Study. https://www.fda.gov/media/155405/download

[34] Burger HU, Gerlinger C, Harbon C, Koch A, Posch M, Rochon J, Schiel A (2021). The use of external controls: To what extent can it currently be recommended?. Pharm Stat.

[35] Ghadessi M, Tang R, Zhou J, Liu R, Wang C, Toyoizumi K, Mei C, Zhang L, Deng CQ, Beckman RA (2020). A roadmap to using historical controls in clinical trials – by Drug Information Association Adaptive Design Scientific Working Group (DIA-ADSWG). Orphanet Journal of Rare Disease.

[36] Wang X, Suttner L, Jemielita T, Li X (2021). Propensity score-integrated Bayesian prior approaches for augmented control designs: a simulation study. J Biopharm Stat.

[37] Best N, Price RG, Pouliquen I, Keene ON (2021). Assessing efficacy in important subgroups in confirmatory trials: An example using Bayesian dynamic borrowing. Pharm Stat.

[38] Lewis CJ, Sarkar S, Zhu J, Carlin BP (2019). Borrowing from historical control data in cancer drug development: a cautionary tale and practical guidelines. Stat Biopharm Res.

[39] Coiffier B, Sarkozy C (2016). Diffuse large B-cell lymphoma: R-CHOP failure-what to do? Hematology 2016. Lymphoma Chall Dir.

[40] Choueiri TK, Fishman MN, Escudier B, McDermott DF, Drake CG, Kluger H, Stadler WM, Perez-Gracia JL, McNeel DG, Curti B, Harrison MR, Plimack ER, Appleman L, Fong L, Albiges L, Cohen L, Young TC, Chasalow SD, Ross-Macdonald P, Srivastava S, Jure-Kunkel M, Kurland JF, Simon JS, Sznol M (2016). Immunomodulatory Activity of Nivolumab in Metastatic Renal Cell Carcinoma. Clin Cancer Res.

[41] Motzer RJ, Tannir NM, McDermott DF, Frontera OA, Melichar B, Choueiri TK, Plimack ER, Barthélémy P, Porta C, George S, Powles T, Donskov F, Neiman V, Kollmannsberger CK, Salman P, Gurney H, Hawkins R, Ravaud A, Grimm MO, Bracarda S, Barrios CH, Tomita Y, Castellano D, Rini BI, Chen AC, Mekan S, McHenry MB, Wind-Rotolo M, Doan J, Sharma P, Hammers HJ, Escudier B, CheckMate 214 Investigators (2018). Nivolumab plus Ipilimumab versus Sunitinib in advanced renal-cell carcinoma. N Engl J Med.

[42] Motzer RJ, Escudier B, McDermott DF, George S, Hammers HJ, Srinivas S, Tykodi SS, Sosman JA, Procopio G, Plimack ER, Castellano D, Choueiri TK, Gurney H, Donskov F, Bono P, Wagstaff J, Gauler TC, Ueda T, Tomita Y, Schutz FA, Kollmannsberger C, Larkin J, Ravaud A, Simon JS, Xu LA, Waxman IM, Sharma P, CheckMate 025 Investigators (2015). Nivolumab versus Everolimus in advanced renal-cell carcinoma. N Engl J Med.

[43] Rini BI, Plimack ER, Stus V, Gafanov R, Hawkins R, Nosov D, Pouliot F, Alekseev B, Soulières D, Melichar B, Vynnychenko I, Kryzhanivska A, Bondarenko I, Azevedo SJ, Borchiellini D, Szczylik C, Markus M, McDermott RS, Bedke J, Tartas S, Chang YH, Tamada S, Shou Q, Perini RF, Chen M, Atkins MB, Powles T, KEYNOTE-426 Investigators (2019). Pembrolizumab plus axitinib versus sunitinib for advanced renal-cell carcinoma. N Engl J Med.

[44] McDermott DF, Lee JL, Ziobro M, Suarez C, Langiewicz P, Matveev VB, Wiechno P, Gafanov RA, Tomczak P, Pouliot F, Donskov F, Alekseev BY, Shin SJ, Bjarnason GA, Castellano D, Silverman RK, Perini RF, Schloss C, Atkins MB (2021). Open-label, single-arm, phase II study of pembrolizumab monotherapy as first-line therapy in patients with advanced non-clear cell renal cell carcinoma. J Clin Oncol.

[45] Hutson TE, Lesovoy V, Al-Shukri S, Stus VP, Lipatov ON, Bair AH, Rosbrook B, Chen C, Kim S, Vogelzang NJ (2013). Axitinib versus sorafenib as first-line therapy in patients with metastatic renal-cell carcinoma: a randomised open-label phase 3 trial. Lancet Oncol.

[46] Motzer RJ, Robbins PB, Powles T, Albiges L, Haanen JB, Larkin J, Mu XJ, Ching KA, Uemura M, Pal SK, Alekseev B, Gravis G, Campbell MT, Penkov K, Lee JL, Hariharan S, Wang X, Zhang W, Wang J, Chudnovsky A, di Pietro A, Donahue AC, Choueiri TK (2020). Avelumab plus axitinib versus sunitinib in advanced renal cell carcinoma: biomarker analysis of the phase 3 JAVELIN Renal 101 trial. Nat Med.

[47] U.S. Food and Drug Administration. 2022 Kymriah Prescribing Information

[48] U.S. Food and Drug Administration. 2022 Yescarta Prescribing Information

